# Temporal Changes in the Cerebrospinal Fluid Level of Hypocretin-1 and Histamine in Narcolepsy

**DOI:** 10.1093/sleep/zsw010

**Published:** 2017-02-09

**Authors:** Régis Lopez, Lucie Barateau, Elisa Evangelista, Sofiene Chenini, Philippe Robert, Isabelle Jaussent, Yves Dauvilliers

**Affiliations:** 1 Unité des Troubles du Sommeil Service de Neurologie, Centre National de Référence Narcolepsie Hypersomnies, Hôpital Gui-de-Chauliac, Montpellier F-34000,France;; 2 Inserm U1061, Montpellier F-34000,France;; 3 Université de Montpellier, Montpellier F-34000,France;; 4 Bioprojet-Biotech, Saint Grégoire F-35760, France

**Keywords:** narcolepsy, cataplexy, hypocretin/orexin, histamine.

## Abstract

**Study Objectives:**

To follow the temporal changes of cerebrospinal fluid (CSF) biomarker levels in narcoleptic patients with unexpected hypocretin level at referral.

**Methods:**

From 2007 to 2015, 170 human leukocyte antigen (HLA) DQB1*06:02-positive patients with primary narcolepsy and definite (*n* = 155, 95 males, 60 females, 36 children) or atypical cataplexy (*n* = 15, 4 males, 3 children) were referred to our center. Cerebrospinal hypocretin deficiency was found in 95.5% and 20% of patients with definitive and atypical cataplexy, respectively. CSF hypocretin-1 (*n* = 6) and histamine/tele-methylhistamine (*n* = 5) levels were assessed twice (median interval: 14.4 months) in four patients with definite and in two with atypical cataplexy and hypocretin level greater than 100 pg/mL at baseline.

**Results:**

CSF hypocretin levels decreased from normal/intermediate to undetectable levels in three of the four patients with definite cataplexy and remained stable in the other (>250 pg/mL). Hypocretin level decreased from 106 to 27 pg/mL in one patient with atypical cataplexy, and remained stable in the other (101 and 106 pg/mL). CSF histamine and tele-methylhistamine levels remained stable, but for one patient showing increased frequency of cataplexy and a strong decrease (−72.5%) of tele-methylhistamine levels several years after disease onset. No significant association was found between relative or absolute change in hypocretin level and demographic/clinical features.

**Conclusions:**

These findings show that in few patients with narcolepsy with cataplexy, symptoms and CSF marker levels can change over time. In these rare patients with cataplexy without baseline hypocretin deficiency, CSF markers should be monitored over time with potential for immune therapies in early stages to try limiting hypocretin neuron loss.

**Statement of Significance**

Definite cataplexy may occur in the absence of hypocretin deficiency in rare cases, especially when close to disease onset. Symptoms and cerebrospinal fluid marker levels can change over time in few patients with narcolepsy with cataplexy that may favor disease heterogeneity. In patients with cataplexy without baseline hypocretin deficiency, cerebrospinal fluid markers should be monitored over time with potential for immune therapies in early stages when the “autoimmune” destructive process is not too advanced to try limiting hypocretin neuron loss.

## INTRODUCTION

Narcolepsy type 1 (NT1), also known as narcolepsy with cataplexy or hypocretin-deficiency syndrome, is a rare chronic sleep disorder characterized by the specific and subtotal loss of hypocretin neurons, which could be immune mediated.^1,2^ Hypocretin-1 (HCRT-1) level in the cerebrospinal fluid (CSF) of patients with NT1 is typically reduced to low or undetectable levels already at diagnosis. Conversely, only 10–20% of patients with narcolepsy without cataplexy have low CSF HCRT-1 levels at diagnosis.^2,3^ A brain study of two patients with narcolepsy without cataplexy showed partial loss of hypocretin neurons, suggesting that the presence of cataplexy in narcolepsy could be linked to the severity of hypocretin neuron loss.^4^ In children with NT1, disease onset is often rapid and dramatic, with the full clinical picture of excessive daytime sleepiness (EDS) and cataplexy occurring within several days.^5^ Conversely, a progressive appearance of NT1 symptoms is frequent in adults. Moreover, clinical follow-up of adult patients with narcolepsy without cataplexy showed that 10% developed cataplexy many years after EDS onset.^6^ A slow appearance of NT1 symptoms together with a progressive decline of CSF HCRT-1 from intermediate to low levels has been recently described in an adult patient.^7^ However, the natural history of hypocretin neuron loss and CSF HCRT-1 level reduction remains unclear.

Here, we report the results of the repeated assessment of CSF HCRT-1 levels in six patients with narcolepsy symptoms and unexpected CSF HCRT-1 level (>100 pg/mL) at baseline. Moreover, it has been reported that the number of histamine (HA) neurons is increased in the tuberomammillary nucleus in patients with NT1,^8,9^ without changes in CSF HA or tele-methylHA (t-MHA) levels.^10^ To our best knowledge, no study has described the temporal changes of CSF HA and t-MHA levels in narcolepsy. Therefore, CSF HA/t-MHA levels were also monitored in five of these patients.

## METHODS

Between 2007 and 2015, 155 patients (95 males and 60 females; 36 children) with definite cataplexy and 15 patients (4 males and 11 females; 3 children) with possible/atypical cataplexy were referred to the Reference National Center for Narcolepsy of Montpellier, France. Definite or possible/atypical cataplexy was defined by the presence of episodes of muscle weakness triggered by strong emotions.^11–13^ Possible or atypical cataplexy was determined based on the medical history and the presence of at least one of the following signs: infrequent episodes (< 1 episode per year), absence of typical triggering factors (for adults only), long duration (>2 min), altered consciousness, unilateral localization, or spontaneous resolution. All patients were HLA DQB1*06:02 positive and narcolepsy was not secondary to another medical condition. Polysomnography (PSG) and multiple sleep latency test (MSLT) recordings performed according to AASM guidelines and CSF HCRT-1 levels were available for all patients.

All patients with definite cataplexy had low CSF HCRT-1 levels (<110 pg/mL, including 80 patients with undetectable levels), with the exception of four subjects (2.6%) with normal (>200 pg/mL) and three (1.9%) with intermediate HCRT-1 levels (between 110 and 200 pg/mL). All patients with narcolepsy with possible/atypical cataplexy (eg, rare and long episodes) had normal CSF HCRT-1 levels, but three patients had low HCRT-1 level (including one patient with undetectable level and two with levels between 100 and 110 pg/mL). Due the unexpected results at referral (baseline measurement), CSF HCRT-1 level was measured twice in four of the seven patients with definite cataplexy and normal/intermediate CSF HCRT-1 level, and in two patients with possible/atypical cataplexy and CSF HCRT-1 level between 100 and 110 pg/mL. All patients gave their informed consent to take part in the study, which was approved by the local ethics committee (Montpellier-France).

All patients had a lumbar puncture between 05:00 and 07:00 pm, and CSF samples were stored immediately at −80°C until the measurement of HCRT-1, HA, and t-MHA levels. HCRT-1 levels were determined in duplicate for each CSF sample by the standard validated direct I^125^ radioimmunoassay (RIA) (Phoenix Pharmaceuticals, Belmont, CA) with an intra-assay variability less than 10% and an inter-assay variability between 20% and 30%.^14^ CSF HCRT-1 levels lower than 110 pg/mL were considered as low, between 110 and 200 pg/mL as intermediate, and above 200 pg/mL as normal. All values were back-referenced to the Stanford reference samples (HHMI Stanford University Center for Narcolepsy, Palo Alto, CA).^14^ CSF HA and t-MHA levels were determined in duplicate using a previously described method through derivatization of primary amines using 4- bromobenzenesulfonyl chloride and subsequent analysis by reversed-phase liquid chromatography with mass spectrometry detection.^10,15^ Intra-assay coefficient variations were 10.6% for HA and 8.1% for t-MHA, and inter-assay coefficient variations were 10.9% for HA and 11.0% for t-MHA.^15^

Spearman’s rank order correlations were used to determine associations between continuous variables. The Kruskall–Wallis test was used to compare continuous and categorical variables with more than two categories. The significance level was set at *p* < .05. Statistical analyses were performed using SAS, version 9.4 (SAS Institute, Cary, NC).

## RESULTS

The clinical, neurophysiological, and biological data of the six patients with lumbar puncture performed twice (median interval of 14.4 months [range 8.5–24.2]) are described in Table 1.

**Table 1:** Demographic, Clinical, and Biological Characteristics of Patients with Narcolepsy.

Variables	Case #1	Case #2	Case #3	Case #4	Case #5	Case #6
Gender	F	F	F	F	F	F
HLA DQB1*alleles	06:02/06:02	06:02/03:01	06:02/06:04	06:02/03:01	06:02/02:01	06:02/03:01
H1N1 vaccination	No	Pandemrix®	No	No	No	No
Age at EDS onset	10	39	16	25	16	30
Age at cataplexy onset	10	39	16	32	26	40
Baseline evaluation						
Age	10	40	17	45	27	54
Disease duration (month)	2	20	21	262	147	288
BMI (kg/m^2^)	20.3	28.7	17.1	37.9	20.8	31.2
Cataplexy frequency	>1/day	>1/day	<1/year	<1/month	3 lifetime	>1/month
Cataplexy phenotype	Definite, total	Definite, partial, and total	Atypical, partial	Definite, partial	Atypical, partial	Definite, partial
Hypnagogic hallucinations	Yes	Yes	Yes	No	Yes	No
Sleep paralysis	Yes	Yes	Yes	No	Yes	No
ESS	ND	18	14	19	17	23
MSL (min)	3.6	12	4.4	3.8	10.0	3.4
SOREMP (PSG/MSLT)	1/3	0/0	0/4	1/5	1/4	0/1
HCRT-1 level (pg/mL)	282	163	106	182	101	250
HA level (pM)	412	287	279	918	220	ND
t-MHA level (pM)	971	732	349	1438	3139	ND
Follow-up evaluation						
Interval between samplings (month)	18	10	21	8	24	10
BMI (kg/m^2^)	27.4	29.7	17.1	32.8	20.2	29.7
Treatment	MPH 50 mg/day	No	MPH 40 mg/day	SXB 9 g/day	MOD 400 mg/day MPH 20 mg/day	No
Cataplexy frequency	>1/day	>1/day	No more cataplexy	>1/week	>1/week	>1/year
ESS	ND	21	7	12	12	23
HCRT-1 level (pg/mL)	<10	<10	27	<10	106	289
HA level (pM)	317	321	177	1088	193	ND
t-MHA level (pM)	1109	585	651	1163	865	ND

HLA = human leukocyte antigen; EDS = excessive daytime sleepiness; BMI = body mass index; ESS = Epworth sleepiness scale (for adults or the version adapted to children); MSL = mean sleep latency; ND = not done; SOREMP = sleep onset rapid eye movement periods; PSG = polysomnography; MSLT = multiple sleep latency test; HCRT = hypocretin; HA = histamine; t-MHA = tele-methylhistamine; MPH = methylphenidate; MOD = modafinil; SXB = sodium oxybate.

### Patient 1

**Figure 1:**
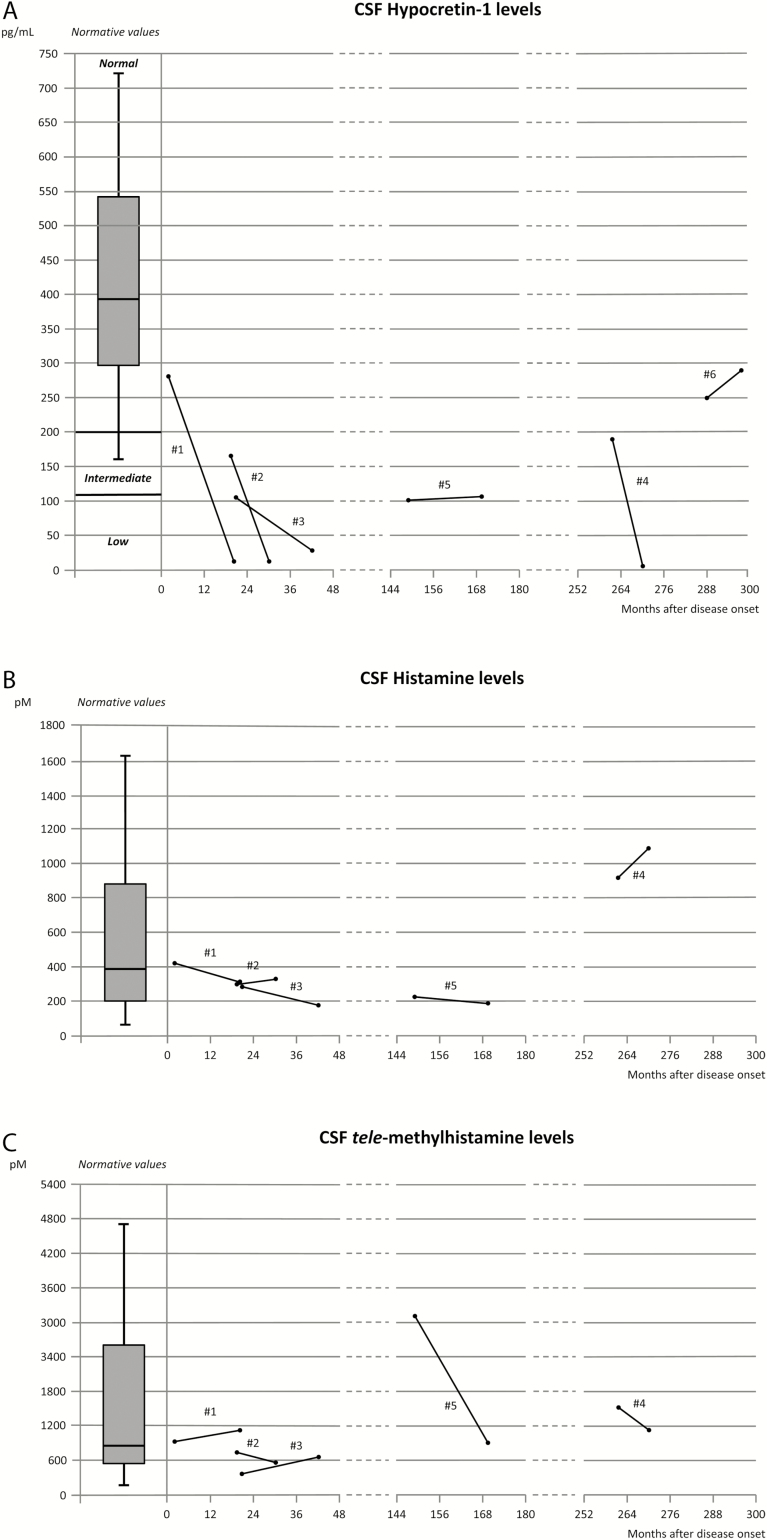
Change in CSF hypocretin-1 (A), histamine (B), and tele-methylhistamine (C) levels from baseline to the second measurement in patients with narcolepsy.

A 10-year-old girl from Martinique (one of the French West Indies islands) presented with the typical clinical phenotype of NT1. EDS started at the age of 10 without any triggering factor and with the appearance of definite cataplexy 1 month later. She also reported vivid nightmares, hypnagogic hallucinations, and sleep paralysis. PSG-MSLT, performed 1 month later, revealed one nocturnal sleep onset rapid eye movement period (SOREMP) and a mean sleep latency of 3.6 min with three SOREMPs. She was homozygous for HLA DQB1*06:02. CSF HCRT-1 level, which was normal (282 pg/mL) at referral (1 month after cataplexy onset), became undetectable (<10 pg/mL) at the second evaluation carried out 18 months later. Baseline CSF HA and t-MHA levels were 412 and 971 pM, respectively, and remained stable (317 and 1109 pM) at the second measurement (Figure 1).

### Patient 2

A 40-year-old Caucasian woman developed daily and definite cataplexy at the age of 39, few months after vaccination with the Pandemrix® vaccine. Three months after cataplexy onset, she described the occurrence of severe EDS, hypnagogic hallucinations, and sleep paralysis. Baseline PSG-MSLT and CSF HCRT-1 measurements were performed at referral (20 months after cataplexy onset) and showed normal mean sleep latency (12 min) without any SOREMP and intermediate CSF HCRT-1 level (163 pg/mL). Due to symptom persistence, PSG-MSLT and CSF sampling were repeated 10 months later in drug-free conditions. PSG-MSLT showed a mean sleep latency of 8 min, five SOREMPs, including one at nighttime. CSF HCRT-1 level was undetectable (<10 pg/mL). CSF HA and t-MHA levels remained stable (287 and 732 pM at baseline and 321 and 585 pM at the second CSF sampling, respectively).

### Patient 3

A 17-year-old girl from Martinique presented with EDS that had started at the age of 16. Two months later she developed atypical partial cataplexy that became rapidly infrequent (<1/year) and was never triggered by emotional factors, such as laugh or surprise. PSG-MSLT performed 21 months after EDS onset showed a mean sleep latency of 4.4 min with four SOREMPs. CSF HCRT-1 level was 106 pg/mL. At the age of 19, treatment with methylphenidate alone was introduced to control EDS. A second CSF sampling carried out at this time showed that HCRT-1 level was decreased to 27 pg/mL while she had no more cataplexy episodes. CSF HA level was also slightly reduced compared with baseline (from 279 to 177 pM), while t-MHA level increased from 349 to 651 pM.

### Patient 4

A 45-year-old Caucasian woman was addressed to our unit at the age of 45 after a car accident caused by EDS. She reported EDS onset at the age of 25 and the appearance of infrequent (<1/month) but definite partial cataplexy 7 years later. At referral, PSG-MSLT recording showed nocturnal SOREMP with a mean sleep latency of 3.8 min and five SOREMPs. CSF HCRT-1 level was intermediate (182 pg/mL) with high HA (918 pM) and t-MHA (1438 pM) levels. Few months after the baseline assessment, cataplexy frequency increased in the absence of any particular event. Treatment with sodium oxybate led to incomplete resolution of cataplexy. A second lumbar puncture performed 8 months after the baseline measurement showed undetectable CSF HCRT-1 level (<10 pg/mL) with almost stable CSF HA (1088 pM) and t-MHA (1163 pM) levels.

### Patient 5

A 27-year-old Caucasian woman presented with EDS, frequent hypnagogic hallucinations, and sleep paralysis since the age of 17, with only three episodes of neck muscle weakness triggered by laugh at the age of 26. PSG-MSLT recording revealed nocturnal SOREMP, mean sleep latency of 10 min, and five SOREMPs. CSF HCRT-1 level was 101 pg/mL. Despite treatment with modafinil and methylphenidate, cataplexy severity and frequency progressively increased to more than one episode per week. A second CSF sampling performed 24 months later showed stable HCRT-1 levels (106 pg/mL). Conversely, CSF t-MHA level was strongly decreased (from 3139 pM at the first sampling to 865 pM), while HA level remained stable (220 and 193 pM).

### Patient 6

A 54-year-old Caucasian woman presented with severe EDS since the age of 30. She reported the occurrence of monthly episodes of partial, definite cataplexy, without hypnagogic hallucinations or sleep paralysis, since the age of 40. PSG-MSLT performed twice in drug-free conditions with an interval of 8 months showed a mean sleep latency of 2.8 min without any SOREMP, and then of 3.4 min with one SOREMP. Baseline (at the age of 54) CSF HCRT-1 level was normal (250 pg/mL). Treatment with modafinil reduced EDS. Cataplexy frequency also decreased to one episode per year. One year later, a third PSG-MSLT recording carried out 2 weeks after modafinil withdrawal showed a mean latency of 5.6 min with one SOREMP. CSF sampling on the same occasion indicated that HCRT-1 level was stable (289 pg/mL). CSF HA or t-MHA levels were not measured.

A strong CSF HCRT-1 reduction was observed in four of the six patients, including three in whom the first CSF sampling was carried out within 2 years after disease onset (Figure 1A). No significant association was found between the relative or absolute change in CSF HCRT-1 level and age, age at EDS or cataplexy onset, cataplexy frequency, and interval between CSF samplings and disease duration. HA levels at the first testing were heterogeneous (median: 287 pM, range: 220–918 pM), but remained quite stable over time (Figure 1B). Similarly, t-MHA levels at baseline were also heterogeneous (median: 970.7 pM, range: 349–3139 pM) and remained stable during the follow-up, with the exception of patient 5 (Figure 1C). No significant association was found between relative or absolute changes in CSF HA or t-MHA levels and age at EDS or cataplexy onset, cataplexy frequency, CSF HCRT-1 levels, and interval between CSF samplings and disease duration. A negative correlation was found only between age at baseline and CSF HA, but not t-MHA, levels (*r* = −0.90; *p* = .04).

## DISCUSSION

We report the results of the repeated CSF HCRT-1 measurement in six of the ten patients with unexpected baseline HCRT-1 results (>100 pg/mL) among all patients (*n* = 170) with EDS and definite or possible/atypical cataplexy at our narcolepsy reference center. At the second evaluation, CSF HCRT-1 concentration was reduced to undetectable levels (<10 pg/mL) in three patients, to 27 pg/mL in one patient, and remained stable in the two others. In contrast, CSF HA levels remained stable, whereas CSF t-MHA level strongly decreased only in one patient.

Although hypothesized in animal studies,^16^ a temporal and causal association between HCRT deficiency and NT1 onset has been reported in only two patients with narcolepsy. The first study showed an abrupt decline from normal to low CSF HCRT-1 levels at disease onset at the age of 10 after exposure to Pandemrix®.^17^ The second study described the slow appearance of NT1 symptoms in parallel with the progressive decrease of CSF HCRT-1 levels down to definite HCRT-1 deficiency in a 45-year-old adult.^7^

Here, we found that definite cataplexy, the pathognomonic symptom of narcolepsy, may occur in the absence of CSF HCRT-1 deficiency (patients 1, 2, 4, and 6), even in the context of the HLA DQB1*06:02 genotype and when narcolepsy is not secondary to another medical condition. Moreover, the second CSF HCRT-1 measurement indicates that levels may decrease over time. On the other hand, in patients 5 and 6, CSF HCRT-1 levels remained stable with levels at 101–106 pg/mL for patient 5 with atypical rare cataplexy, and greater than 200 pg/mL at both assessments for patient 6 with baseline definitive cataplexy. To confirm these findings, we re-measured the CSF HCRT-1 levels in the last four available samples in duplicate in a single RIA assay using a similar pooled sample as an internal standard solution. We found mean intra- and inter-assay coefficient variations of 7% and 28%, respectively. These results confirmed the low intra-assay and intermediate inter-assay variabilities as already reported for RIA hypocretin quantification,^18^ likely in relation with the variability in antibody batch quality, purity of the peptide, and specific activity (counts per minute per one mole of hypocretin) of the labeled I^125^-hypocretin ligand. HA and t-MHA were also quantified in duplicate using a previously specific, sensitive, robust, and validated method.^15^ Altogether, our measurements confirmed that quantifications of HCRT-1, HA, and t-MHA are reproducible including intra- and inter-assay variability in agreement with the criteria outlined in the regulatory bioanalysis guidances (US and European).

HCRT neuron loss progression may vary among patients with narcolepsy, in agreement with the disease heterogeneity. The occurrence of EDS and cataplexy symptoms and disease progression are often different in drug-free patients with narcolepsy with abrupt or progressive onset. Among the population of 155 patients with definite cataplexy, cataplexy occurred before sleepiness in 3.5% of them (mean delay between 1 and 5 years), within the same year in 67.5%, and cataplexy started after sleepiness onset in 29% (mean delay >3 years). Moreover, EDS and cataplexy symptoms may show some fluctuations during the follow-up. This could be linked to different pathogenic mechanisms based on the patient response to environmental stimuli (ie, infection or vaccination). A severe and abrupt NT1 onset has often been reported in children, whether triggered by Pandemrix® vaccination or not.^5,19^ In contrast, a more progressive development is usually described in adults, with often several months or years between EDS and cataplexy onset.^20^ Indeed, follow-up assessment of narcoleptic patients without cataplexy showed that 10% developed cataplexy many years after EDS onset.^6^ The mechanism underlying the slow occurrence of narcoleptic symptoms remains unknown. We hypothesize that a progressive or multiple-hit process targets HCRT neurons, depending on the different immune response to the underlying triggers (for instance streptococcal and H1N1 infections, Pandemrix®) in patients with highly predisposing genetic background (such as HLA class I and II, T-cell receptors alpha, P2RY11).^21^ This leads first to partial neuron loss, as described in NT2 (ie, without cataplexy),^4^ and then to almost 90% of cell loss, as often described in NT1 (ie, with cataplexy).^22,23^ However, we cannot exclude that other neurotransmitters might be involved in aggravating or compensating HCRT neuron loss and disease symptoms. For instance, patient 5 displayed increased frequency of cataplexy together with a strong decrease (−72.5%) of t-MHA levels several years after disease onset. A relationship between cataplexy and HA activity has been already described in animal models of narcolepsy^23,24^ and in humans, where significant cataplexy improvement is observed upon treatment with an inverse agonist of the HA receptor H3.^25^

Moreover, in two patients with cataplexy at baseline (3 and 6), cataplexy episodes became rare or absent in drug-free conditions, while HCRT decreased to very low levels only in patient 3. Such spontaneous improvement in cataplexy frequency and intensity over time has been previously described in drug-free adults and children with HCRT deficiency, suggesting that besides HCRT, additional players are also involved.^5,26^ This kind of clinical improvement is often reported in the course of autoimmune disorders, showing partial remission after an abrupt acute onset phase.^27^ Moreover, some patients with narcolepsy may cope with, or even avoid triggering factors to prevent cataplexy recurrence; however, this cannot entirely explain cataplexy fluctuations over time.^1^ Several neurotransmitters are certainly involved in compensatory mechanisms to regulate cataplexy severity.^13^

This study has some limitations. First, we cannot exclude some potential inter-assay variability in CSF biomarker measurements, with values obtained between 10% and 28%. Despite the use of standard validated techniques of measurements of HCRT-1, HA, and t-MHA following the guidelines, the variabilities found may lower the clinical significance of our results. Second, we may acknowledge the differences in disease duration at baseline evaluation and in the interval between the two CSF measurements. Third, although cataplexy was assessed by physicians with a strong expertise in narcolepsy, its diagnosis was based on the patient’s medical history and recall bias cannot be excluded. Moreover, the definition of cataplexy is not very precise and the distinction between definite and possible/atypical cataplexy is poorly codified.^11–13^ Therefore, we advocate precise guidelines for cataplexy assessment based on home or in-laboratory video recording to be further validated by narcolepsy experts.

To conclude, we report that definite cataplexy may occur in the absence of CSF HCRT-1 deficiency especially when close to disease onset, even in the context of the HLA DQB1*06:02 genotype and when not secondary to another medical condition. HCRT neuron loss progression may vary among patients with narcolepsy, in agreement with the disease heterogeneity. Our findings may highlight the potential for immune-based therapies in early stages to save HCRT neurons, especially when the “autoimmune” destructive process is not too advanced (ie, patients with intermediate or normal CSF HCRT-1 levels at diagnosis).^28,29^ Finally, we suggest that CSF biomarkers should be repeatedly evaluated in the case of cataplexy in the absence of CSF HCRT-1 deficiency at baseline.

## FUNDING

This was not an industry-supported study. Support for this research was provided by the French Ministry of Research and Higher Education, Project Agence Nationale de la Recherche-2014-ImmunitySleep, and Aviesan-ITMO 2014—BioNarcoImmunity.

## DISCLOSURE STATEMENT

YD has been an invited speaker and consultant for UCB Pharma, JAZZ, and BIOPROJET. PR is an employee of Bioprojet-Biotech. Other coauthors had no conflict of interest.
